# Image Segmentation of Brain MRI Based on LTriDP and Superpixels of Improved SLIC

**DOI:** 10.3390/brainsci10020116

**Published:** 2020-02-20

**Authors:** Yu Wang, Qi Qi, Xuanjing Shen

**Affiliations:** 1 College of Applied Technology, Jilin University, Changchun 130012, China; 2 College of Computer Science and Technology, Jilin University, Changchun 130012, China; qiqi15@mails.jlu.edu.cn (Q.Q.); xjshen@jlu.edu.cn (X.S.)

**Keywords:** superpixel segmentation, 3D histogram reconstruction, simple linear iterative clustering, local tri-directional pattern

## Abstract

Non-uniform gray distribution and blurred edges often result in bias during the superpixel segmentation of medical images of magnetic resonance imaging (MRI). To this end, we propose a novel superpixel segmentation algorithm by integrating texture features and improved simple linear iterative clustering (SLIC). First, a 3D histogram reconstruction model is used to reconstruct the input image, which is further enhanced by gamma transformation. Next, the local tri-directional pattern descriptor is used to extract texture features of the image; this is followed by an improved SLIC superpixel segmentation. Finally, a novel clustering-center updating rule is proposed, using pixels with gray difference with original clustering centers smaller than a predefined threshold. The experiments on the Whole Brain Atlas (WBA) image database showed that, compared to existing state-of-the-art methods, our superpixel segmentation algorithm generated significantly more uniform superpixels, and demonstrated the performance accuracy of the superpixel segmentation in both fuzzy boundaries and fuzzy regions.

## 1. Introduction

With the rapid development of medical imaging technology, medical images play an increasingly important role in clinical diagnosis and treatment. The medical image segmentation technique, as the basis of medical image applications, has been broadly used in various medical research areas and practices, such as CAD computer aided diagnosis (Computer Aided Diagnosis), IGS (Imaging Guided Surgery), and ORT (Operative Radiation Therapy). There are multiple kinds of medical imaging techniques, such as CT (Computed Tomography), MRI (Magnetic Resonance Imaging), ultrasound imaging, and PET (Positron Emission Tomography). Among them, MRI is a kind of tomography that uses the magnetic resonance phenomenon to obtain electromagnetic signals of the human body, and accordingly, reconstruct the human body.

MRI outperforms other existing techniques due to the following advantages: (1) it causes no ionizing radiation damage to the human body; (2) it can freely choose the required profile by adjusting the magnetic field, therefore, providing more abundant image information to define the nature of the lesion; (3) it can detect the dynamic changes of water contained in the tissue more sensitively as compared to CT, rendering a several times higher resolution for soft tissue to detect the tissue lesions. With the aforementioned advantages, MRI has been widely applied to various imaging diagnoses of different organs of the whole body, demonstrating an increasingly important role in medical diagnosis.

Magnetic resonance imaging is increasingly being used to assess brain growth and development in infants. Such studies are often based on quantitative analysis of anatomical segmentations of brain MR images. However, the large changes in brain shape and appearance associated with development and the lower signal-to-noise ratio and partial volume effects in the neonatal brain present challenges for automatic segmentation of neonatal MR imaging data. The segmentation algorithm that models the intensities across the whole brain by introducing a structural hierarchy and anatomical constraints achieves highly accurate results and is very robust across a wide range of gestational ages [1]. Currently, the brain tumor segmentation method can automatically detect and extract whole tumors from 3D-MRI. The algorithm is based on a hybrid approach that relies on a brain symmetry analysis method that combines region-based and boundary-based segmentation methods. In addition, the segmentation system has been tested and evaluated on 3D-MRIs of various subjects with different tumor types and shapes [2].

However, drawbacks still exist: First, the gray distribution of the MRI image is not uniform. Second, the imaging mechanism of the MRI and the complexity of the human body make it difficult to extract the target area accurately, with its edge in the image often blurred, resulting in bias during the clinical diagnosis and creation of the clinical treatment plan. Moreover, moving organs such as hearts and intestines may also cause artifacts in the image. So, improving the accuracy of MRI image segmentation has become a research hotspot [3]. 

## 2. Related Work

Image segmentation is a common image processing step in many computer vision applications with the purpose segmenting pixels into different classes. Salim Lahmiri et al. [4] compared three automated diagnosis systems to detect glioma in brain magnetic resonance images (MRIs). As improved variants of particle swarm optimization (PSO) algorithms, the fractional-order Darwinian particle swarm optimization (FODPSO) and Darwinian particle swarm optimization (DPSO) were proposed for image segmentation. They also compared the segmentation performance of PSO, DPSO, and FODPSO as parametric approaches to existing methods, namely the parametric fuzzy c-means (FCM) algorithm and the non-parametric Otsu segmentation technique, by applying the different techniques to five biomedical images. The obtained experimental results showed that particle swarm-based algorithms outperformed both FCM and Otsu segmentation techniques [5]. However, these methods easily fall into a local optimum due to the lack of dynamic adjustment of speed, which leads to low convergence accuracy and difficulty in convergence. In addition, it is difficult to control the parameters, so it is necessary to make strategies to choose the right parameters to achieve the best results.

The traditional image segmentation method takes the pixel as the basic unit. However, with rapid development of the medical imaging technology, the current images contain far more pixels, but also include diverse additional information, such as temporal and spatial relationships between pixels. Consequently, the segmentation method, which only relies only on the pixel, fails to take into account the additional information, and has been unable to meet the requirements of accurate medical diagnoses. To solve this problem, the superpixel was proposed for the first time by Ren et al. [6] and has become a hot research topic [7].

Beyond the pixel in the image, texture on the surface of all kinds of objects is another important feature, and has been widely used in the study of image segmentation. For example, Yang et al. [8] proposed a texture-based image segmentation method in 2012, which used the color and texture of pixels as features, followed by a least squares support vector machine (LS-SVM) classifier to perform the image segmentation. In 2014, Shen et al. [9] proposed an interaction time and texture feature-based segmentation method, which first calculated the probability of each pixel through a lazy random walk (LRW) algorith, and learned superpixel edge and segmentation through optimizing an energy function defined on the interaction time and texture features.

In 2015, Min et al. [10] proposed a level set model by combining intensity and texture to segment complex natural images. The method uses a global segmentation algorithm to capture the intensity information of the image, and then uses an adaptive scale local variation algorithm to extract the texture features. Finally, the extracted intensity and texture are both applied to the level set to segment the image. In 2017, Xiao et al. [11] proposed a superpixel segmentation algorithm based on iterative and adaptive clustering. In the proposed algorithm, color, contour, texture, and spatial features are all taken into account, and the weights of different features are automatically learned by content perception to meet the needs of various image instances.

Differing from pixels and texture, the superpixel is a set of pixels sharing similar characteristics that could better describe the underlying structure of an image by extracting its local features [6,12]. Through merging pixels, the number of superpixels in the image is largely decreased, which greatly reduces the burden of subsequent processing and, therefore, improves the efficiency. Owing to these advantages, the superpixel has become an important part of computer vision algorithms and has been widely studied [13,14,15,16].

In medical image processing, an image is often first segmented into several superpixels in preprocessing, thereby facilitating the subsequent steps. In 2015, Tian et al. [17] proposed a superpixel-based 3D image segmentation algorithm, and applied it to the prostate MRI images. This method uses superpixels as the basic unit to construct a 3D image, and then uses a graph cutting algorithm to segment the prostate region via minimizing an energy function. 

To summarize the above, the superpixel and its derived texture features play an important role in medical image segmentation, and various related methods have been proposed and applied to MRI image processing. However, the accuracy of superpixel segmentation is still not satisfactory, which directly affects the quality of subsequent operations and the accuracy of target extraction. 

In order to further improve the accuracy of superpixel segmentation, this paper, on the basis of a simple linear iterative clustering (SLIC) algorithm and refenence [18], proposes an improved simple SLIC superpixel segmentation algorithm for medical images based on texture features. First, we use the 3D histogram reconstruction model to reconstruct the gray level of the input image and enhance the target area of the reconstructed image through gamma enhancement. Next, we use the magnitude of the local tri-directional pattern (LTriDP) algorithm to extract the texture features of the image. Finally, we use the improved SLIC superpixel segmentation algorithm to generate the segmentation results. The experiments showed that the proposed algorithm can achieve better results of medical image superpixel segmentation when compared with the other existing methods.

## 3. The Proposed Method

**Figure 1:**
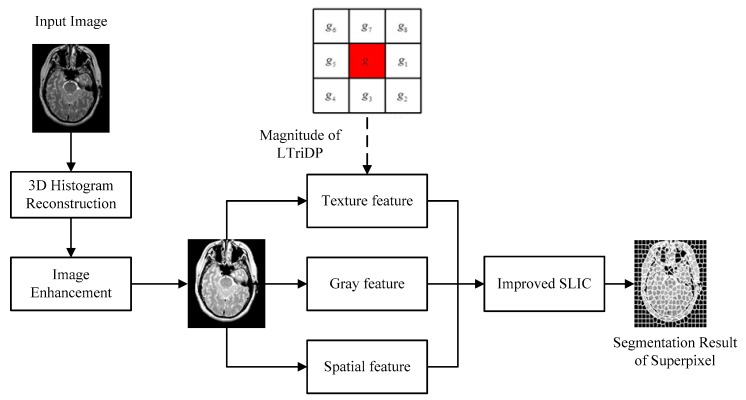
The framework of the proposed algorithm. LTriDP: local tri-directional pattern; SLIC: simple linear iterative clustering.

In order to solve the problems of blurred edge and non-uniform gray distribution in the process of superpixel segmentation of brain MRI images, an improved SLIC superpixel segmentation algorithm is proposed in this paper. It outperforms the other existing state-of-the-art methods owing to the following contributions: (1) the 3D histogram reconstruction model proposed in reference [19] is used to reconstruct the gray level of brain MRI images; (2) the target region of the image is enhanced by gamma transformation proposed in reference [20], and the texture features of the image can be represented by the magnitude of LTriDP; and (3) superpixel segmentation is carried out by an improved SLIC algorithm. In the process of iteratively updating the clustering center by SLIC, only the pixels whose gray difference with the original clustering center is less than α are used to calculate the final image segmentation results. The experiments on the Whole Brain Atlas (WBA) image database showed that the proposed superpixel segmentation algorithm generated significantly more uniform superpixels and demonstrated the performance accuracy of the superpixel segmentation in both fuzzy boundaries and fuzzy regions, as compared to the other methods. The framework of the proposed algorithm is shown in Figure 1.

### 3.1. 3D Histogram Reconstruction

Due to the image formation and the complexity of the human body’s structure, the non-uniform gray distribution can affect the quality of MRI images, which will mislead the doctors’ judgment of the microstructure of images and then affect the medical diagnosis. Therefore, it is very important to reconstruct the gray values of medical images. In this paper, a 3D histogram reconstruction model proposed in reference [19] is used to reconstruct the source image.

The main idea of this method is to use a 3D histogram to represent the image, three coordinate axes to represent the gray value of the pixels *f*(*x*,*y*), the mean value *g*(*x*,*y*) of the 3 × 3 neighborhood, and the median value *h*(*x*,*y*) of the 3 × 3 neighborhood. For an image with uniform gray scale, the triples (*f*(*x*,*y*), *g*(*x*,*y*), *h*(*x*,*y*)) should be distributed along the diagonal direction of the volume in the 3D histogram.

**Figure 2:**
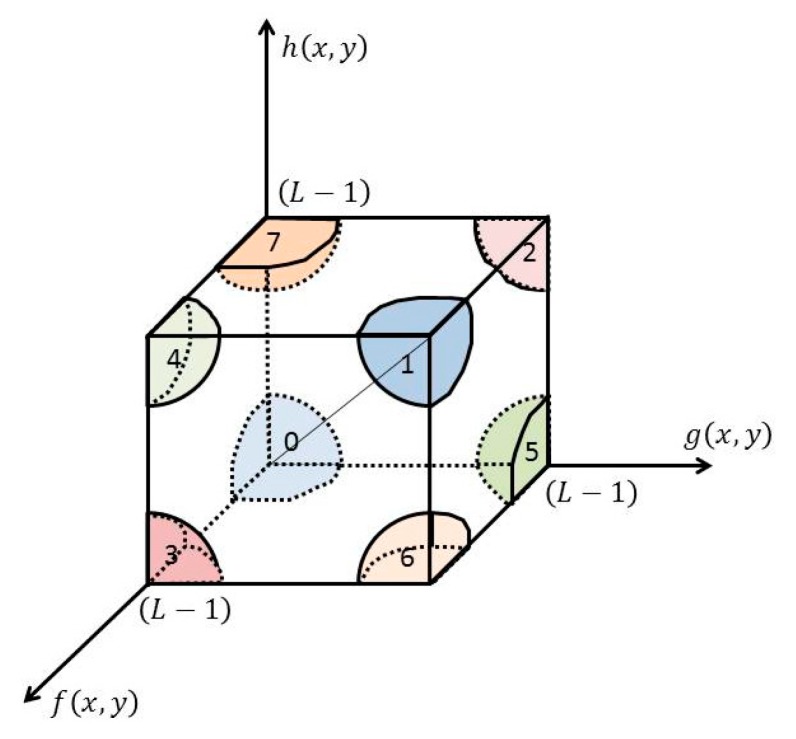
The model of 3D histogram reconstruction.

Because of the limitation of imaging equipment and the interference of the human body, some pixels will be distributed outside the volume diagonal of the 3D histogram. As shown in Figure 2, the 3-D histogram reconstruction model divides the 3D histogram into eight regions (0–7) for processing.

Region 0 and Region 1: The pixels in these two regions are normally distributed pixels without correction.
Region 2 and Region 3: The gray values of pixels in these two regions are corrected by means of mean and median values as follows:
  (1) $$f^{*}=\left(g+h\right)/2$$
Region 4 and Region 5: The mean values of pixels in these two regions are corrected by means of gray values and median values as follows:
  (2) $$g^{*}=\left(f+h\right)/2$$
Region 6 and Region 7: The gray values and mean values of pixels in these two regions are corrected by median values as follows:
  (3) $$f^{*}=g^{*}=h^{*}$$

In the corrected 3D histogram, the pixels are all distributed near the volume diagonal. The gray reconstructed medical image can be obtained as follows:

(4) $$f\left(x,y\right)=\left(f^{*}+g^{*}+h^{*}\right)/3$$

### 3.2. Image Enhancement

Due to the limitation of the imaging mechanism of the medical image, the target organization in MR images is darker, especially at the edge of the target area. Because of the low contrast with the background gray value, the target area is hard to segment, resulting in inaccurate final segmentation results. In order to highlight the edges of the target area in medical images and facilitate subsequent operations, the image should be enhanced before superpixel segmentation, and the γ enhancement proposed in reference [20] is used in this paper.

**Figure 3:**
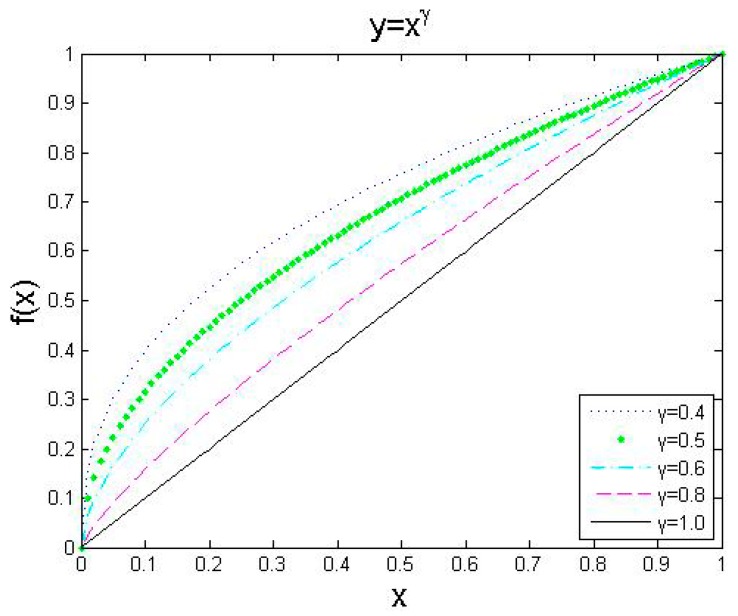
The curves of γ enhancement with different parameters.

The exponential equation with exponential γ is used to enhance the variables, and the range of the variables is [0, 1]. We set γ to 0.4, 0.5, 0.6, 0.8, and 1.0 and the enhancement curves are shown in Figure 3. The enhancement equation for the enhancement of MR images is as follows:

(5) $$I^{\prime }\left(x,y\right)=255{\left(\frac{I\left(x,y\right)}{255}\right)}^{\gamma }$$

where *I*(*x*,*y*) is the source image, and *I*’(*x*,*y*) is the enhanced image. The value of γ is set to 0.5 in this paper.

### 3.3. Magnitude of LTriDP

Owing to the peculiarity and complexity of medical images, there is no universal method for extracting texture features. However, texture features of medical images can reflect the important information of organs and tissues in images, which is beneficial to the improvement of the accuracy of superpixel segmentation of medical images. Local binary pattern (LBP) codes have the advantages of rotation invariance and gray scale invariance [21]. They are suitable for medical images, which contain valuable irregular texture features.

LTriDP is an extension of LBP codes [18]. In addition to considering the relationship between the center pixel and the neighborhood pixels, as LBPs do, they also consider the relationship between neighborhood pixels. Therefore, this paper uses the magnitude of LTriDP to extract texture features. In order to reduce the computational complexity of the texture feature extraction algorithm, the pixels in the eight neighborhoods of the central pixel point are selected to create the magnitude of LTriDP. The differences between the central pixel *g_c_* and its neighboring pixels *g_i_*, and the differences of gray values between *g_i_* and its nearest neighboring pixels in the vertical or horizontal direction are calculated and are used to calculate *M*_1_, *M*_2_ in the magnitude of LTriDP. The calculation process is as follows:

(6) $$\{\begin{array}{c} M_{1}=\sqrt{{\left(g_{8}-g_{c}\right)}^{2}+{\left(g_{i+1}-g_{c}\right)}^{2}}\\ M_{2}=\sqrt{{\left(g_{8}-g_{i}\right)}^{2}+{\left(g_{i+1}-g_{i}\right)}^{2}} \end{array}\begin{array}{cc} & if \end{array}\;i=1$$

(7) $$\{\begin{array}{c} M_{1}=\sqrt{{\left(g_{i-1}-g_{c}\right)}^{2}+{\left(g_{i+1}-g_{c}\right)}^{2}}\\ M_{2}=\sqrt{{\left(g_{i-1}-g_{c}\right)}^{2}+{\left(g_{i+1}-g_{i}\right)}^{2}} \end{array}\begin{array}{cc} & if \end{array}\;i=2,3,\cdots ,7$$

(8) {M1=(gi−1−gc)2+(g1−gc)2M2=(gi−1−gi)2+(g1−gi)2 ifi=8

Then, a magnitude value is assigned to eight neighborhood pixels by the Equation (9) based on the calculated *M*_1_ and *M*_2_:

(9) $$Mag(i)=\{\begin{array}{c} 1,M_{1}\geq M_{2}\\ 0,M_{1}<M_{2} \end{array}$$

The local texture features of central pixel *g_c_* can be calculated as follows:

(10) $$LTriDP_{mag}(g_{c})=\{Mag(1),Mag(2),\cdots ,Mag(8)\}$$

(11) $$LTriDP(g_{c})=\sum_{i=0}^{7}2^{i}\times LTriDP_{mag}(g_{c})$$

### 3.4. Superpixel of Improved SLIC

Simple linear iterative clustering (SLIC) is an efficient superpixel generation algorithm that uses k-means clustering to reduce the search range [22]. Compared with other superpixel segmentation algorithms, SLIC algorithm is efficient in storage, fast in computing time, low in computational complexity and fitness to edge, which improves the performance of image segmentation. It meets the requirements of medical image segmentation for accuracy, so the SLIC superpixel segmentation algorithm is suitable for segmentation of medical images.

SLIC clusters the pixels according to the color similarity and spatial distance of the pixels, which is an iterative clustering process. In reference [23], it is pointed out that in the SLIC superpixel segmentation of color images, some pixels will be misclassified after the first iteration. All the pixels belonging to one class will be used to update the clustering center, and these misclassified pixels will have an impact on the update process. After several iterations, the errors will be magnified, which will affect the final result of the superpixel segmentation. Inspired by reference [23], only the pixels with similar gray value to the original clustering center are used to update the clustering center in this paper. The steps of improved SLIC superpixel segmentation are as follows:

**Step 1**: *K* clustering centers are initialized with a span of $S=\sqrt{N/K}$, where *N* is the number of pixels in the image. Initialize the label of each pixel *i* belonging to the class l(i)=−1, and the distance between each pixel and the cluster center with d(i)=∞.

**Step 2**: For each *i*-th pixel in the 2*S* × 2*S* neighborhood of each cluster center *C_k_*, the distance between *i* and *C_k_* is calculated by the following equations:

(12) $$d_{c}=\sqrt{{\left(g_{C_{k}}-g_{i}\right)}^{2}}$$

(13) $$d_{s}=\sqrt{{\left(x_{C_{k}}-x_{i}\right)}^{2}+{\left(y_{C_{k}}-y_{i}\right)}^{2}}$$

(14) $$d_{t}=\sqrt{{\left(t_{C_{k}}-t_{i}\right)}^{2}}$$

(15) $$D=\sqrt{{\left(\frac{d_{c}}{N_{c}}\right)}^{2}+{\left(\frac{d_{s}}{N_{s}}\right)}^{2}+{\left(\frac{d_{t}}{N_{t}}\right)}^{2}}$$

where *d_c_* is the distance of gray, *d_s_* is spatial distance, *d_t_* is the distance of texture features, *N_c_* is the maximum of gray-scale distance, $N_{s}=S=\sqrt{N/K}$ is the maximum space distance within the class, and *N_t_* is the maximum of texture feature distance. If *D* < *d*(*i*), then *d*(*i*) = *D*,*l*(*i*) = *k*.

**Step 3**: Update the cluster center. The improved SLIC calculates the *CO_k_* and *S_k_* of new cluster centers *C_k_* as follows:

(16) $$CO_{k}=\frac{1}{N_{k}}\sum_{i\in \Omega_{k}}CO_{i}$$

(17) $$S_{k}=\frac{1}{N_{k}}\sum_{i\in \Omega_{k}}S_{i}$$

(18) $$\Omega_{k}=\left(\left|l_{k}-l_{i}\right|<\alpha \right)\cap G_{k}$$

where *G_k_* is the cluster set of pixels denoted as the center *C_k_*, *N_k_* is the number of pixels in the set of *G_k_*, and *CO_k_* and *S_k_* represent the means of gray and distance of the pixels, respectively, which take *C_k_* as the center. Step 2 and Step 3 iterations are executed until a predetermined number of iterations are reached. In general, the ideal superpixel can be obtained after 10 iterations. Here, α is the standard deviation of image gray and *l_i_* is the gray value of pixel *i*.

In summary, after pre-processing, we extract the LTriDP texture feature and then the improved SLIC is used to realize the final superpixel segmentation. The 3D histogram reconstruction was used to reconstruct the gray values of medical images, which can be treated as a pre-processing method. Gamma correction has a larger response output for smaller input values, while for larger input values, the response output increases by a small margin. It is suitable for enhancing medical images. This value is usually obtained by experience. Here, the gamma value is 0.5, as in reference [20]. The descriptor of LTriDP was used to extract texture features from 2D images. Super pixels are generated by means of k-means clustering in SLIC, which produce the best result on image boundaries on the Berkeley benchmark. Here, we improved the updating process of the clustering center so only the pixels that have similar gray values to the original clustering center are used to update the clustering center.

## 4. Experiment and Analysis

### 4.1. Database and Configuration

There are many kinds of medical image databases used for image segmentation. The Whole Brain Atlas (WBA) image database was used as the experimental database in this paper, which is currently the most widely used medical image segmentation database [24]. The brain medical image database is collected by Harvard Medical School, and the URL is http://www.med.harvard.edu/aanlib/home.html.

**Figure 4:**
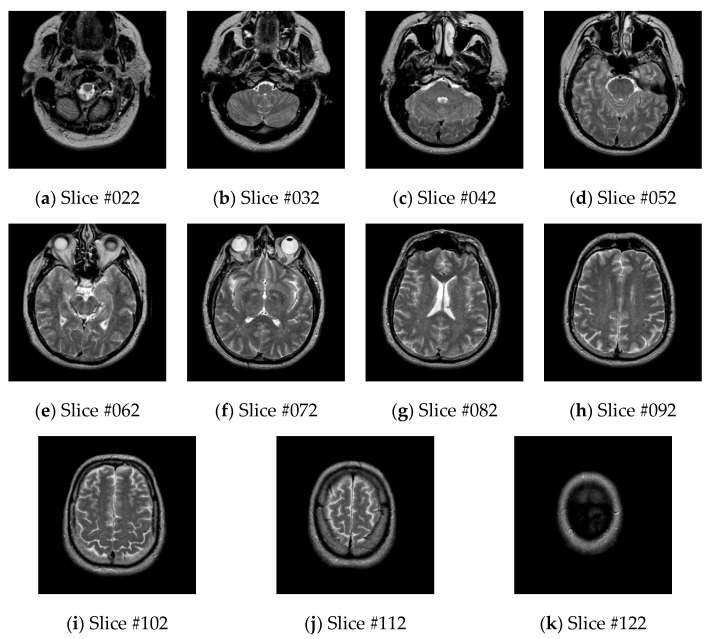
MR-T2 brain transaxial images.

The WBA image database contains three different types of brain images: (1) transaxial images, (2) sagittal images, (3) coronal images. Each type of image contains five different modes of brain images: MR-T1 images, MR-T2 images, FDG-based PET images, PET-MR-T1 fusion images, and PET-MR-T2 fusion images. There are 126 MR-T2 brain transaxial images, and the slices #022, #032, #042, #052, #062, #072, #082, #092, #102, #112, and #122 are often used as a dataset for medical image segmentation [22], as shown in Figure 4.

### 4.2. Evaluation Criteria 

Due to the complexity of medical images, the superpixel blocks in the results of superpixel segmentation are mixed, and it is difficult to see the advantages of this algorithm intuitively. Therefore, two quantitative evaluation indicators, undersegmentation error and boundary match, were used to evaluate the experimental results in this paper [25].

*UE* (Undersegmentation Error) is used to measure the “overflow” to the outside of the ground truth region in the superpixel that overlaps with the ground truth region. Assuming that the superpixel segmentation algorithm divides the image into superpixels $s_{1},s_{2},\cdots ,s_{n}$, undersegmentation error is defined as the proportion of the part outside the target area in the superpixel to the whole target area, which can be expressed as:

(19) $$UE=\frac{\left[\sum_{\left\{s_{i}|s_{i}\cap g\neq \phi \right\}}s_{i}\right]-g}{g}$$

The size of the target area is used to normalize the value of the “overflow” pixels, and the range of *UE* is [0, 1]. The smaller the *UE* value, the smaller the “overflow” to the outside of the ground truth in the segmentation result, and the more accurate is the segmentation result.

BM (Boundary Match) depends on the definition of boundary, which is used to measure the proportion of the overlap between the boundary of the superpixel segmentation result and the boundary of the target area in ground truth. The equation is as follows:

(20) $$BM=\frac{SP\left(img\right)\cap GT\left(img\right)}{SP\left(img\right)}$$

where *SP*(*img*) is the boundary of image superpixel segmentation, and *GT*(*img*) is the boundary of the target area marked in the ground truth of the image. The value range of *BM* is [0, 1], the larger the value of BM, the more parts of the boundary the segmentation results coincide with in the boundary of ground truth, and the more accurate is the segmentation result.

### 4.3. Experiment Results and Analysis

**Figure 5:**
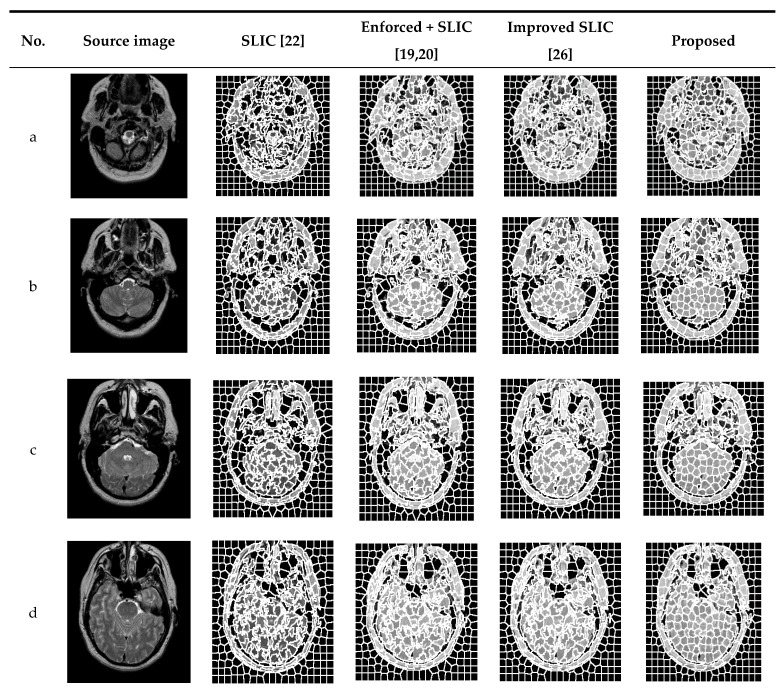

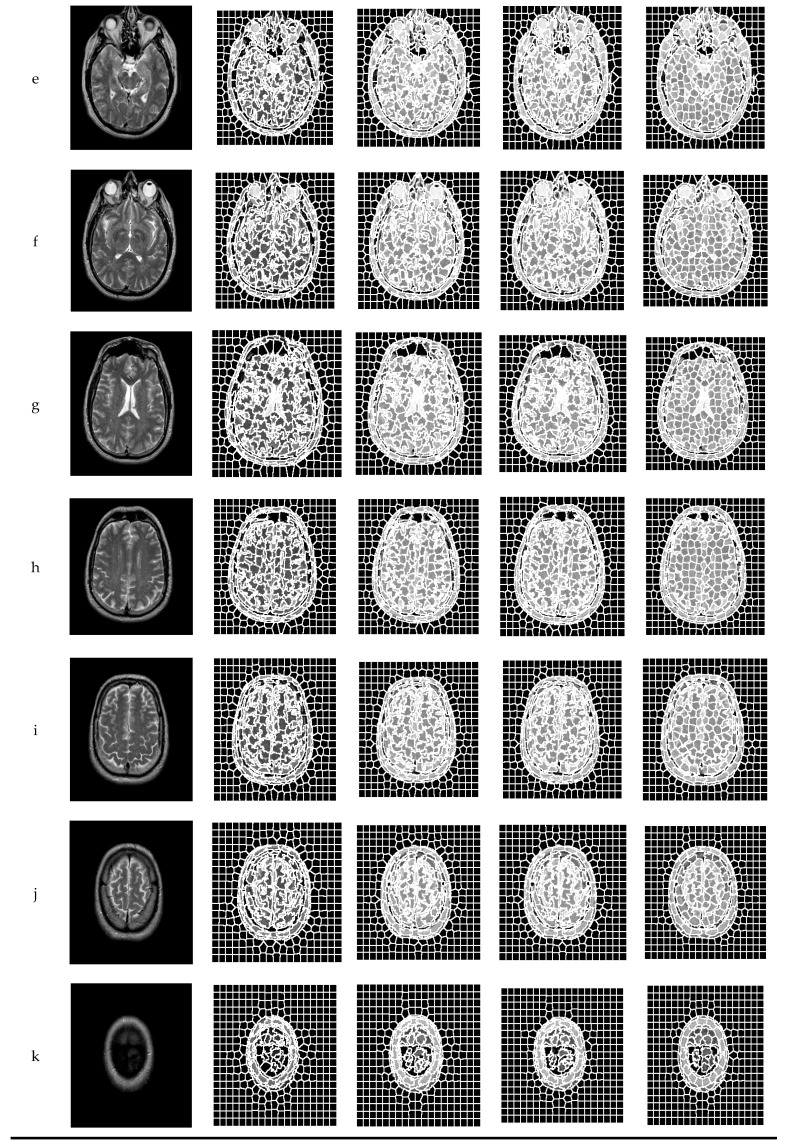
The segmentation results of different algorithms.

In order to verify the effectiveness of the proposed algorithm, experiments were carried out on several MRI brain images and were compared with traditional SLIC and enforced + SLIC algorithms. Figure 5 shows the segmentation results of traditional SLIC, enforced + SLIC, improved SLIC, and the proposed algorithm in this paper. As shown in Figure 5, after preprocessing, the target areas that are close to the color of the background area are segmented, the gray value consistency of the superpixel blocks is better, and the segmentation results are more accurate.

Compared with SLIC, improved SLIC greatly improved the accuracy of superpixel segmentation and the consistency of superpixel blocks. As can be seen from Figure 5, after adding the local texture features of the image, the image features are better when calculating the similarity between pixels, and the pixels grouped into the same superpixel have more similar features. The superpixel edge of the proposed algorithm coincides with the edge of the target area in the image. The target areas close to the gray value of the background area and the smaller target areas that are misclassified are also significantly reduced. The superpixel blocks are more uniform and regular, and the accuracy of superpixel segmentation and the consistency in the superpixels are further improved in the proposed algorithm.

**Figure 6:**
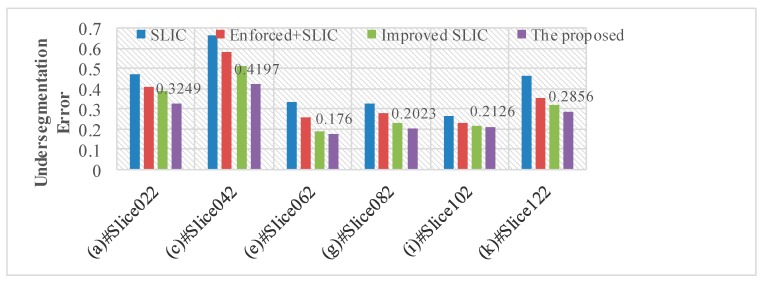
The Undersegmentation error of different algorithms on selected slices.

The comparison of undersegmentation error of different algorithms is shown in Table 1 and Figure 6. As can be seen from Table 1, after preprocessing, the part of the image that is misclassified is significantly reduced. In the improved SLIC algorithm, only the pixels whose gray difference between the original clustering centers is less than α were used to update the clustering center. So, the gray value of the pixels in the superpixel is more consistent, and the edge of the superpixel is closer to the edge of the target area. When the “overflow” part of the target area is smaller, it results in the segmentation being more accurate.

**Figure 7:**
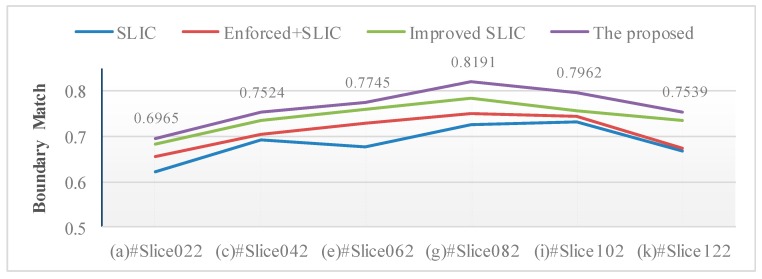
The boundary match of different algorithms on selected slices.

The comparison of the boundary match of different algorithms is shown in Table 2 and Figure 7. The BM value of the proposed algorithm segmentation results is further improved compared with other algorithms. The edge of the proposed algorithm segmentation results fits the ground truth and the segmentation result is more accurate.

As can be seen from the results above, the results of superpixel segmentation was significantly improved after adding texture features. Compared with other algorithms, BM was greatly improved and UE was significantly reduced, which indicates that the segmentation of the partial volume effect is more accurate. After adding texture information to the improved SLIC algorithm, the pixels clustered in the same superpixel have more similar features. So, the probability of dividing the edge and the target area into the same superpixel is higher, the edge of the superpixel segmentation result fits the actual target area, and the classification results are more accurate. Therefore, the proposed algorithm in this paper is more suitable for the actual edge of the target area in the superpixel segmentation results of brain MRI medical images. The number of misclassified pixels is significantly reduced, and the results are more accurate.

To define undersegmentation error and boundary match, we used the difference method to find matches between superpixels and ground truth. The difference method is a supervised evaluation method, which evaluates the performance of the segmentation algorithm according to the difference between the actual segmentation results and the standard segmentation results. The pixel method mainly measures the difference between the two by calculating the proportion of the incorrectly classified pixels and the correctly classified pixels in the total number of standard segmentation pixels. MR-T2 images are used widely in brain image segmentation, and they make up a representative data set in the field of image segmentation.

## 5. Conclusions

An improved SLIC superpixel segmentation algorithm based on texture features is proposed in this paper. First, a 3-D histogram reconstruction model is used to reconstruct the image gray level, eliminating the non-uniformity of gray level in the image. Then, after enhancement, the texture feature is extracted by the magnitude of LTriDP codes. Finally, superpixel segmentation is carried out using a SLIC algorithm that improves the iterative process of clustering centers for brain MR images. The superpixels generated by the proposed algorithm are compact and nearly uniform, which is suitable for the requirements of real time and accuracy in medical image segmentation. The experimental results show that the proposed algorithm can segment the blurred edges and regions in the image. The proposed algorithm has lower UE and higher BM. The edges of the segmentation results are more consistent with the standard ground truth edges, and the segmentation results are more accurate. The main innovation of the proposed method is the improvement of superpixels. The experiment tested the efficiency of the proposed method; it still cannot meet the real time and accuracy requirements of clinical scenarios, so that will be the main direction of our future work.

## Figures and Tables

**Table 1:** The comparison of UE of different algorithms.

No.	SLIC [22]	Enforced + SLIC [19,20]	Improved SLIC [26]	Proposed
(a) #Slice022	0.4711	0.4077	0.3848	0.3249
(b) #Slice032	0.6098	0.4766	0.4053	0.3677
(c) #Slice042	0.6632	0.5778	0.5116	0.4197
(d) #Slice052	0.4156	0.3456	0.2919	0.2686
(e) #Slice062	0.3313	0.2599	0.1916	0.1760
(f) #Slice072	0.2841	0.2075	0.1798	0.1659
(g) #Slice082	0.3275	0.2769	0.2305	0.2023
(h) #Slice092	0.3047	0.2136	0.1881	0.1609
(i) #Slice102	0.2655	0.2311	0.2189	0.2126
(j) #Slice112	0.2876	0.2652	0.2343	0.1867
(k) #Slice122	0.4629	0.3554	0.3184	0.2856

**Table 2:** The comparison of the boundary match of different algorithms.

No.	SLIC [22]	Enforced + SLIC [19,20]	Improved SLIC [26]	Proposed
(a) #Slice022	0.6221	0.6565	0.6818	0.6965
(b) #Slice032	0.6209	0.6666	0.7021	0.7337
(c) #Slice042	0.6928	0.7040	0.7362	0.7524
(d) #Slice052	0.6707	0.7182	0.7322	0.7751
(e) #Slice062	0.6780	0.7298	0.7594	0.7745
(f) #Slice072	0.6655	0.6812	0.7255	0.7534
(g) #Slice082	0.7251	0.7502	0.7840	0.8191
(h) #Slice092	0.7334	0.7465	0.7616	0.8224
(i) #Slice102	0.7311	0.7431	0.7571	0.7962
(j) #Slice112	0.7281	0.7592	0.7871	0.8086
(k) #Slice122	0.6668	0.6729	0.7354	0.7539
